# The Role of Community Organisation, Religion, Spirituality and Cultural Beliefs on Diabetes Social Support and Self-Management in Sub-Saharan Africa: Integrative Literature Review

**DOI:** 10.1007/s10943-024-02233-y

**Published:** 2025-01-24

**Authors:** Israel Bekele Molla, Virginia Hagger, Mette Juel Rothmann, Bodil Rasmussen

**Affiliations:** 1 Institute of Health, School of Nursing, Jimma University, Jimma, Ethiopia; 2 The Centre for Quality and Patient Safety, School of Nursing and Midwifery, Faculty of Health, Deakin University, Melbourne, Australia; 3 Steno Diabetes Centre Odense, Odense University Hospital, Odense, Denmark; 4 Department of Clinical Research, Faculty of Health Science, University of Southern Denmark, Odense, Denmark; 5 Department of Public Health, Faculty of Health and Medical Science, University of Copenhagen, Copenhagen, Denmark

**Keywords:** Community organisation, Religion, Spirituality, Cultural beliefs, Social support, Diabetes self-management, Sub-Saharan African countries

## Abstract

To examine the evidence for the role of community organisations, religion, spirituality, cultural beliefs, and social support in diabetes self-management, we undertook an integrative literature review utilising MEDLINE, APA PsycINFO, CINAHL, and grey literature databases. The selected articles were appraised for quality, and the extracted data were analysed thematically. The search yielded 1586 articles, and after eliminating duplicates, 1434 titles and abstracts were screened, followed by a full-text review of 103 articles. Ultimately, 47 articles met the inclusion criteria for the review, utilising various study designs, including qualitative, quantitative, mixed-methods, and nonrandomised clinical trials. These findings indicate that spirituality and religiosity can positively affect diabetes self-management by providing motivation, coping skills, social support, and guidance for healthy behaviours. A strong social support system enhances diabetes self-management and glycaemic control for individuals with diabetes. However, some aspects of religion and culture, such as beliefs about medications, may also pose challenges or barriers to diabetes self-management. Adherence to medication, food choices, physical activity, and the use of complementary or alternative medicine can be influenced by sociocultural factors. Additionally, cultural beliefs and social norms influence understanding diabetes aetiology, management, and symptom reactions. The findings highlight that it is crucial to understand the cultural, religious, or spiritual influences that can either assist or impede self-management habits in individuals with diabetes and could inform interventions that support personalised and effective care.

## Introduction

Diabetes mellitus is a chronic condition that necessitates lifelong dedication to diet, medication, physical activity, blood glucose monitoring, and regular medical check-ups to avoid any long-term complications (Mercer et al., 2019; Mukhtar et al., 2020). Globally, more than half a billion people were estimated to have diabetes in 2021, with more than 80% residing in developing nations (Federation, 2021). However, in Africa, the overall prevalence of diabetes is 4.5% (Federation, 2021). Notably, the rate of known diabetes varies by country. In sub-Saharan Africa, Benin has the lowest prevalence at 0.6%, whereas the highest prevalence is in Réunion, affecting 18.2% of the population (Pastakia et al., 2017).

In sub-Saharan Africa, three out of every five people with diabetes remain undiagnosed, and three out of every four people die before the age of 60 as a result of complications associated with diabetes (IDF, 2019).

Moreover, how individuals take care of their diabetes can vary on the basis of their cultural, spiritual, or religious beliefs, as well as their level of social support (Sari et al., 2022). These beliefs can sometimes hinder optimal self-management, leading to delays in seeking medical help and an overreliance on traditional or religious healers (Iregbu et al., 2022). In addition, cultural beliefs significantly affect how illnesses are understood, detected, and treated and influence an individual’s coping mechanisms and perceptions of managing complex conditions, such as diabetes. Furthermore, these beliefs play a role in shaping how individuals are perceived and treated by their family and community (Arnault, 2018; Osokpo & Riegel, 2021).

Diabetes self-management requires adherence to exercise and diet, but cultural factors can influence these behaviours (Eshete et al., 2023). In various societies, exercise is often disregarded and viewed as a luxury or only part of Western culture, which discourages people from participating in physical activities (Foley & BeLue, 2017; Mogre et al., 2019). Additionally, being overweight is sometimes perceived as a sign of wealth or good health in some cultures, making it challenging for people with diabetes to maintain a healthy weight as part of their diabetes care plan (Masupe et al., 2022).

In this context, community organisation in diabetes management refers to a group or entity within a specific community that actively supports individuals with diabetes through various services, resources, education, and advocacy (Wilson et al., 2012). These organisations often focus on improving diabetes outcomes by facilitating access to healthcare, providing peer support, promoting healthy lifestyles, and advocating for better healthcare policies (Norris et al., 2006). Furthermore, they work closely with healthcare providers, patients, and community members to create a supportive environment that empowers individuals to manage their diabetes effectively (Lubega et al., 2023).

When discussing religion and spirituality in diabetes management, it is important to distinguish between these two concepts. On the other hand, spirituality is a broader and more individual concept that refers to a personal sense of connection to something greater than oneself, which can involve seeking meaning, purpose, and inner peace (Onyishi et al., 2021; Spencer, 2012). Unlike religion, spirituality may or may not be associated with a specific religion and often focuses on personal growth, self-awareness, and inner experiences (Spencer, 2012). Moreover, spirituality is more individualised and less structured than religion, emphasising personal belief systems and practices that may or may not align with formal religious traditions (Lepherd, 2015).

The incorporation of religion and spirituality into diabetes care can enhance overall outcomes by promoting resilience, community, and social support (Berkoh et al., 2022; Onyishi et al., 2021). For example, studies have indicated that individuals who participate in religious activities tend to have lower HbA1c levels and receive emotional support as a coping mechanism (Botchway et al., 2022a, 2022b). Similarly, another study revealed that greater social support and social involvement in diabetes care are associated with better diabetes self-management (Chan et al., 2020; Stanulewicz et al., 2019). In contrast, poor social support, such as inadequate emotional support and a small social network, has been associated with higher hospital admission rates (Ketema et al., 2020; Teshome Tesfaye et al., 2020); suboptimal diabetes self-management (Bonger et al., 2018); and higher rates of depression, blindness, and foot ulcers (Karimy et al., 2018).

Given these considerations, understanding the role of community organisations, religion, spirituality, and cultural beliefs in diabetes self-management in sub-Saharan Africa is crucial, as these factors are deeply embedded in daily life and significantly shape how individuals manage their conditions and access social support. Despite their importance, these factors often receive insufficient attention in medical care, which can undermine efforts to enhance diabetes self-management. The gaps in the literature, such as the need for context-specific interventions, holistic approaches, and an understanding of the efficacy of community support and the interplay of these factors, are essential in diabetes care. Ultimately, exploring these factors can lead to improved health outcomes, more individually tailored healthcare, informed policy development, and greater patient empowerment and engagement. Therefore, the purpose of this review is to determine the role of community organisations, religion, spirituality, cultural beliefs, and social support in self-management among people living with diabetes in sub-Saharan Africa.

## Methods

### Study Design

An integrative literature review approach was used (Toronto & Remington, 2020; Whittemore & Knafl, 2005) to include qualitative, quantitative, mixed methods, and nonrandomised clinical trial designs to gain a deeper understanding of the spiritual, social, and cultural influences on diabetes self-management and social support.

The review encompassed all peer-reviewed studies published in English that examined community organisations, religion, spirituality, cultural beliefs, social support, and diabetes self-management. The studies focused on adults (> 18 years) living with type 1 diabetes (T1DM) or type 2 diabetes (T2DM) in sub-Saharan African countries.

Non-peer reviews, studies involving other types of diabetes, and studies involving people aged less than 18 were excluded. The search was not limited by date. Nevertheless, no pertinent papers were identified before 2003.

The review protocol was registered with the PROSPERO database of systematic reviews, and reporting adhered to the Preferred Reporting Items for Systematic Reviews and Meta-Analyses (PRISMA) guidelines (Page et al., 2021).

### Search Strategy and Information

The search strategy was developed in consultation with a medical librarian. To identify the keywords and descriptors used to index studies related to the themes of this review, a preliminary, unstructured search was conducted via the EBSCO platform via the MEDLINE database. The search terms used Boolean operators AND and OR to develop a search strategy (see Appendix 2), specific to each database: MEDLINE, APA PsycINFO, and CINAHL via EBSCO and EMBASE. The first 20 pages in Google Scholar were screened, and the reference lists of the included studies were hand-searched to identify eligible studies. The search was carried out between April and June 2023.

### Data Selection

**Fig. 1:**
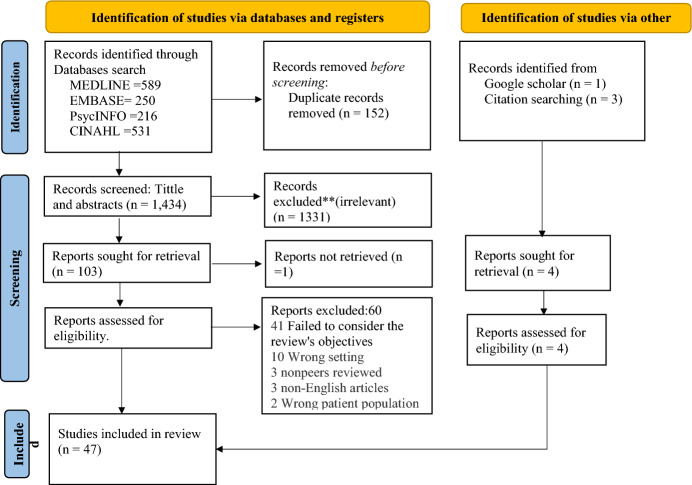
Flowchart for paper identification, screening, and inclusion

The results were exported into the reference management software EndNote (Version 20.1), and duplicate records were removed. The citations were then exported into Covidence software for screening (Veritas Health Innovation, Melbourne, Australia) (Covidence, 2021). Titles and abstracts were screened independently by two reviewers, and any disagreements were resolved by discussing the eligibility criteria. The full texts were screened similarly by the two reviewers. All pertinent articles were thoroughly read, and those that did not fit the requirements for this evaluation were excluded. Figure 1 shows the screening and selection flowchart.

### Data Extraction

The data extracted included title, author(s), publication year, country, study design, aim/research question, target population, inclusion/exclusion criteria, participant number, outcome results, conclusion, recommendation, and limitations. A structured data matrix was employed to organise and analyse the extracted data following systematic review reporting guidelines (Kitchenham, 2004).

### Critical Evaluation of the Included Studies

As we included multiple study designs, evaluation criteria relevant to each study were applied. All included studies were critically appraised for methodological rigour, using checklists from the Joanna Briggs Institute (JBI) for qualitative studies (Lockwood et al., 2015), quantitative studies (Moola, 2020), quasi-experimental studies (nonrandomised experimental studies) (Tufanaru, 2020) and mixed studies (Moorley & Cathala, 2019), as appropriate for the multiple study designs (Toronto & Remington, 2020).

Most of the articles included in this review received excellent quality scores, reflecting the robustness and reliability of the research (Toronto & Remington, 2020). The research teams discussed the scoring criteria, where "yes" was given a score of 1, and "no and not clear" was given a score of 0. The quality assessment process is outlined in Appendix 3 as a supplementary File. Two independent reviewers were involved in the assessment process to increase the accuracy of the evaluation and minimise potential bias. Each reviewer independently evaluated the studies on the basis of predefined criteria, such as the clarity of the research objectives, methodological rigour, appropriateness of the data analysis, and validity of the findings (Toronto & Remington, 2020). After the independent assessments, any discrepancies between the reviewers’ evaluations were discussed and resolved through consensus, further strengthening the credibility of the quality assessment. This rigorous approach ensures that the literature reviewed is both relevant and of high quality, providing a solid foundation for the findings and conclusions drawn in this review (Whittemore & Knafl, 2005).

### Data Analysis and Synthesis

Following the approach outlined by Barnett-Page and Thomas (Barnett-Page & Thomas, 2009), a narrative synthesis was employed to analyse and summarise the findings of the studies. This involved actively engaging with the data through iterative reading and notetaking to identify patterns. An Excel spreadsheet served as a structured data matrix for comparing and analysing data, involving the examination of keywords to discern underlying concepts, aligning with the principles of narrative synthesis described by Barnett-Page and Thomas (Barnett-Page & Thomas, 2009). The results were organised into themes and subthemes, primarily centred around cultural beliefs, spiritual or religious outcomes, and social support. The authors further refined these topics on the basis of similarities, connections, and patterns to ensure a comprehensive synthesis of the information.

## Results

### Study Selection

The search yielded 1586 articles from various databases: 531 from CINAHL, 589 from Medline, 216 from APA PSYCHINFO, 250 from Embase, 3 from the reference lists of included articles and 1 from Google Scholar. After eliminating duplicate entries (n = 152), 1434 titles and abstracts were screened. Among these, 1331 articles were excluded because they did not meet the inclusion criteria. Upon further scrutiny of the 107 full-text articles retrieved, 60 were eliminated because they did not align with the review’s objectives or settings. Ultimately, 47 articles met the inclusion criteria and were included in the review. A visual representation of the selection process is provided in Fig. 1, outlining the reasons for exclusion.

Most of the studies were high quality (scoring > 6/10), as detailed in Appendix 3 as a supplementary, which provides a comprehensive overview of the quality assessment conducted for each included study. Numerous studies in our analysis adopted a cross-sectional design. Given the inherent characteristics of such studies, it is crucial to acknowledge that the cultural, religious, or spiritual perspectives employed by specific authors in interpreting the included studies’ results might have impacted their subjective understanding of the data. This influence could introduce bias into the reporting of outcomes, highlighting the importance of recognising and accounting for these interpretive lenses in the evaluation of study findings.

## Study Characteristics

This review included 47 articles published between 2005 and 2023, including 28 qualitative studies, 16 quantitative studies, two nonrandomised clinical trials, and one mixed-methods study (see Table 1, and Appendix 1). Most of these studies were conducted in 11 of 48 countries in sub-Saharan Africa. Specifically, 12 studies were conducted in Ghana, 10 in South Africa, six in Nigeria, five in Ethiopia, three in Uganda, three in Kenya, three in Senegal, two in Cameroon, one in Benin, one in Malawi and one in Zimbabwe (see Table 2, and Appendix 1).

**Table 1:** Summary of included study designs examining the role of community organisation, religion, spirituality, and cultural beliefs in diabetes social support and self-management in sub-Saharan Africa

Category of the studies	Number of studies
Qualitative studies	28
Quantitative studies	16
Clinical trial (Non-RCT) study	2
Mixed study	1
Total	47

**Table 2:** Summary of study location that explores the role of community organisation, religion, spirituality, and cultural beliefs in diabetes social support and self-management in sub-Saharan Africa

Location of studies	Number of studies
Ghana	12
South Africa	10
Nigeria	6
Ethiopia	5
Uganda	3
Kenya	3
Senegal	3
Cameroon	2
Benin	1
Malawi	1
Zimbabwe	1
Total	47

All studies included participants of both genders aged 18 years and above.

Twelve studies incorporated questionnaires or standardised tools for data collection (Adejoh, 2014; Affusim & Francis, 2018; Botchway et al., 2022a, 2022b; Botchway et al., 2021; Botchway et al., 2022a, 2022b; Jaafaripooyan et al., 2021; Lugaya et al., 2022; Olagbemide et al., 2021; Olorunfemi & Ojewole, 2018; Onyango et al., 2022; Osuji et al., 2018; Shilubane et al., 2016). Additionally, one mixed methods study utilised both interviews and standardised instruments (Berkoh et al., 2022), with the remaining studies utilising in-depth interviews or focus group discussions.

Only two studies focused specifically on participants with type 1 diabetes (Visagie et al., 2018; Willemse et al., 2018). The remaining studies involved participants with type 2 diabetes only or a combination of both types. The predominant focus was on individuals with diabetes, although three studies also included primary healthcare providers (Lloyd et al., 2023; Masupe et al., 2022; Mogre et al., 2019). Additionally, three studies encompassed a broader perspective by involving families, caregivers, friends, or peer support groups (Atinga et al., 2018; Baumann et al., 2015; BeLue et al., 2018).

Collectively, the studies included 5,964 individuals. Among these, 5,743 were individuals diagnosed with diabetes, 48 were healthcare professionals, 133 were family and caregivers, and 40 were community members.

**Fig. 2:**
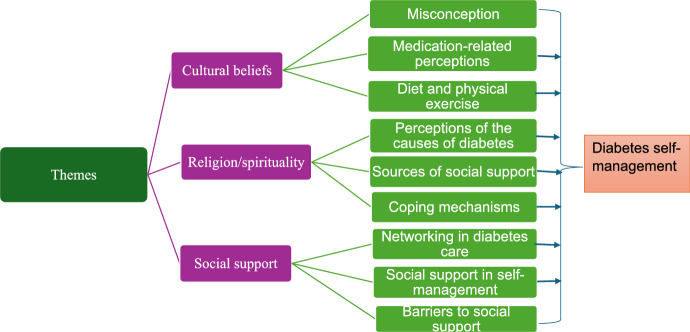
Illustrates the connections between themes and subthemes that influence diabetes self-management in sub-Saharan Africa

Figure 2 clearly illustrates the main themes and subthemes identified from the study findings on diabetes self-management. The findings are organised into three distinct themes: cultural beliefs related to diabetes self-management, the role of religion and spirituality in diabetes management, and the impact of social support on diabetes self-management. Each theme is further divided into three subthemes, which highlight the nuanced factors that influence diabetes care. The hierarchical tree illustrated in Fig. 2 emphasises the interconnectedness of each theme and subtheme, as well as their unique contributions to shaping diabetes self-management.

## Cultural Beliefs and Diabetes Self-Management

The review examined the role of cultural beliefs in diabetes management, revealing three prominent subthemes: (1) misconception, (2) medication-related perceptions, and (3) diet and physical exercise.

### A. Misconceptions

Numerous misconceptions regarding the causes, signs, and symptoms of diabetes are often influenced by social, cultural, behavioural, and linguistic factors within specific communities. These misconceptions may prompt individuals with diabetes to seek care from alternative sources, such as herbalists, local remedies, and spiritual healers, rather than going to medical facilities (Mogre et al., 2019). As a result of their perceptions, they prefer herbal medications such as saltwater, bitter vegetables, and local plants created by native doctors. These remedies are believed to neutralise and dilute blood glucose levels, with individuals placing faith in their potency not only for curing but also for preventing potential illness triggers (Tusubira et al., 2021).

Furthermore, misconceptions can lead to exclusion and stigma for people with diabetes (PWD), making it harder for them to effectively manage their blood glucose levels through lifestyle modification and following dietary recommendations. For example, some individuals with diabetes may feel compelled to avoid community organisations and social gatherings where they cannot consume the offered food, leading to social isolation and emotional distress. As one individual stated, “…When I do not eat their food, they start gossiping about me. As a result, I stopped attending community meetings.” In prompt of emotional coping, “…they do blame me for my diabetes” (PWD, Lloyd, page 7). This stigma can also prompt emotional coping mechanisms, as people with diabetes might face blame for their condition, leading to nondisclosure of their diabetes to avoid further judgement (Lloyd et al., 2023; Masupe et al., 2022).

Fear of stigmatisation due to their condition often causes individuals with diabetes to hesitate to share their diagnosis with friends, family, and close relatives. This reluctance negatively impacts their self-management behaviours and follow-up at clinics, as they avoid seeking medical care to prevent further stigmatisation (Mogre et al., 2019).

### B. Medication-Related Perceptions

Many people with diabetes in Ethiopia, Nigeria, and Uganda believe that healthcare practitioners only partially understand their condition. Consequently, some patients reject or modify their medications in search of a cure. In efforts to increase the likelihood of curing diabetes, some patients combine alternative treatments with conventional biomedicine, hoping for increased effectiveness or a complete cure (Agofure et al., 2018; Awah et al., 2008; Bayked et al., 2022).

The Sociocultural context significantly impacts medication-related perceptions of PWD. For example, insulin treatments, in particular, are linked to heightened severity, as patients believe that insulin is prescribed when their diabetes does not respond to other treatments; this perception is accompanied by a fear that insulin could harm or poison their body (Habte et al., 2017b; Olorunfemi & Ojewole, 2018). Moreover, cultural norms in these regions discourage individuals with diabetes, asserting that certain morbidities, including diabetes, are trivialised, viewed as a normal part of life, or not considered a serious disease. Consequently, using medication solely for the treatment of diabetes is deemed against the norm, is seen as unnecessary, and is perceived as a waste of time and money (Atinga et al., 2018; De-Graft Aikins, 2005).

In Cameroon, people with diabetes also exhibited negative attitudes towards medicinal prescriptions, reflecting broader scepticism towards biomedicine in the region (Awah et al., 2008).

### C. Diet and Physical Exercise

In Benin, food access and information about local food selection and recipes are linked to cultural perceptions and norms, especially concerning fruits and vegetables, because vegetables and fruits are considered commodities of the poor (Alaofè et al., 2021; Foley & BeLue, 2017). Similarly, in Ghana and South Africa, cultural factors affect how people with diabetes follow dietary recommendations and make decisions about their food choices and preparations. Interestingly, being overweight is often seen as a symbol of wealth and achievement, whereas losing weight is not always viewed positively, which influences self-management (Mogre et al., 2019; Pienaar & Reid, 2021). Furthermore, some individuals with diabetes in Ghana and Senegal may not exercise because they believe it is a luxury only for wealthy individuals or is simply part of Western society (Foley & BeLue, 2017; Mogre et al., 2019). In addition, in Cameroon and Nigeria, some people with diabetes may be hesitant to lose weight because of concerns about how it will impact their social image, and there is also a stigma surrounding weight loss and HIV/AIDS (Awah et al., 2008; Olorunfemi & Ojewole, 2018).

## Religion or Spirituality and Self-Management

This review examined how religion and spiritual practices can help people with diabetes in sub-Saharan Africa. Three subthemes were constructed: (1) perceptions of the causes of diabetes, (2) sources of social support, and (3) coping mechanisms.

### A. Perceptions About the Causes of Diabetes

In many studies, diabetes is believed to be caused by supernatural influences, such as fate, punishment from God, spiritual disruption, sorcery, witchcraft, or family spells. Additionally, the review highlighted that traumatic life events were a way to rationalise the cause of the condition, and people believed that diabetes was treated through the improvement of one’s spiritual or divine connection (Abdulrehman et al., 2016; Agofure et al., 2018; Aikins, 2005; Atinga et al., 2018; Bayked et al., 2022; Berkoh et al., 2022; Hjelm & Mufunda, 2010; Mogre et al., 2019; Shilubane et al., 2016). As a result, patients may give up on their self-management, believing that divine forces cause diabetes forces, it must also be treated through mystical means. This belief can lead to a focus on appeasing God or gods rather than adhering to effective management techniques. Moreover, patients may mistakenly believe that treatment conflicts with their religious or spiritual beliefs, hindering their ability to engage in self-management and negatively impacting patient outcomes (Alaofè et al., 2021; Atinga et al., 2018; Bayked et al., 2022; Habte et al., 2017a). Another serious issue in sub-Saharan Africa is the abuse of patients in faith-based community organisations such as spiritual prayer camps, especially people with diabetes, who are accused of causing their condition through bad behaviour and sinful actions. In these camps, patients may face physical mistreatment and isolation and are often required to pay fees to pastors for fasting and prayers, which can create a financial burden on already economically strained individuals and families (Korsah, 2023; Korsah & Domfeh, 2020). This financial strain is particularly challenging for individuals with diabetes, who may already be struggling with the cost of medical care and medications.

### B. Source of Social Support

Religious leaders, community members, and faith-based community organisations, therefore, serve as the primary sources of social support for people with diabetes by encouraging communication and spiritual counselling. These groups can offer support networks, prevention and management programs, resources, and information on diabetes prevention, management, and risk factors. Additionally, they can assist with necessities, such as food, shelter, medical services, and financial aid (Ameyaw Korsah & Ameyaw Domfeh, 2020; Lloyd et al., 2023; Mphwanthe et al., 2021). For example, in Malawi, religious communities and prayers are seen as helpful in achieving weight loss and adopting healthy eating habits through fasting and prostration (Mphwanthe et al., 2021).

### C. Coping Mechanisms

In many settings, following a diabetes diagnosis, faith-based community organisations such as prayer, communion, spiritual healing, fasting, and holy water serve as coping mechanisms that help patients control their blood glucose levels and physical health. These spiritual practices are essential in supporting patients in overcoming the difficulties and stress associated with their diabetes (Alaofè et al., 2021; Bayked et al., 2022; Lloyd et al., 2023; Rotheram-Borus et al., 2012; Tabong et al., 2018). This review revealed that increased participation in religious activities correlated with reduced HbA1c levels (Botchway et al., 2022a, 2022b). Furthermore, people with diabetes often rely on hope and faith to manage their condition. This can motivate them to seek medical attention and follow the advice of healthcare professionals, as they have faith that God can heal their diabetes through the care of doctors and nurses (Korsah, 2023; Korsah & Domfeh, 2020).

## Social Support and Diabetes Self-Management

The review revealed that social support significantly influenced various aspects of life. Three subthemes were constructed: (1) networking in diabetes care, (2) social support in self-management, and (3) barriers to social support in self-management.

### A. Social Networking in Self-Management

Networking with friends, relatives, adult children, spouses, and community members, community organisations such as religious bodies, and social clubs is the main source of social support in diabetes management (Tabong et al., 2018; Willemse et al., 2018). In particular, building strong friendships creates a supportive environment that is associated with reduced HbA1c (Visagie et al., 2018). In addition, family members in several sub-Sahara African countries, such as Ghana, Nigeria, Senegal, South Africa, Uganda, and Zimbabwe, play a vital role in helping patients manage their diabetes. They offer essential support in various aspects, such as medication management, monitoring meal preparation, monitoring blood glucose levels, scheduling doctor appointments, managing stress, creating a supportive environment, and encouraging exercise and lifestyle adjustments (Affusim & Francis, 2018; BeLue et al., 2018; Hjelm & Mufunda, 2010; Mphasha et al., 2022; Tabong et al., 2018; Tusubira et al., 2021).

### B. Social Support in Self-Management

Strong family support and financial assistance for medical expenses were found to be significantly linked to improved self-management and glycaemic control in individuals with type 2 diabetes in Kenya, Nigeria, Senegal, and Uganda (Belue et al., 2012; Lugaya et al., 2022; Olagbemide et al., 2021; Onyango et al., 2022; Osuji et al., 2018). Moreover, peer support for people with type 2 diabetes significantly reduces fasting blood glucose, cholesterol, body mass index, and diastolic pressure, according to nonrandomised control trials in Uganda and Cameroon. **In addition to these physiological benefits,** diabetes self-management behaviours, such as healthy eating, medication adherence, exercise, and emotional well-being, also improve significantly (Assah et al., 2015; Baumann et al., 2015).

Furthermore, in Benin, community organisations, neighbourhood support and group dynamics encouraged people with type 2 diabetes to participate and engage in physical activity (Alaofè et al., 2021). Interestingly, a hospital-based study from Ghana reported that kin composition and household composition significantly impacted social support, but social support did not significantly affect glycaemic control (Botchway et al., 2021). However, among people with diabetes in South Africa, social support was positively related to self-management practices for diabetic meal planning, foot care, physical activity, blood glucose testing and the ability to handle participants’ feelings about living with diabetes (Werfalli et al., 2020).

### C. Barriers to Social Support in Self-Management

Community organisations and family systems play a crucial role in daily diabetes management decisions in sub-Saharan Africa. However, family support can also be a source of stress, particularly if family members do not understand the condition or do not provide adequate support. This dynamic is significant because daily diabetes management decisions such as what to eat, how often to eat, how to interact with others, and how much money to allocate for diabetes management are all influenced by family systems (Belue et al., 2012). For example, in Malawi, various obstacles, such as attending social events that involve food, conflicting dietary advice, and inadequate family support, can make it difficult for individuals to manage their type 2 diabetes effectively (Mphwanthe et al., 2021).

## Discussion

The optimal treatment of diabetes requires an understanding of how community organisations, culture, religion, and spirituality affect self-management and social support. To our knowledge, this is the first extensive review that collects and examines empirical studies on the role of community organisations, cultural, religious, and spiritual factors in self-management and social support among people with diabetes in sub-Saharan countries. The findings indicate that these factors generally have both positive and negative influences on self-management.

Our review revealed that cultural beliefs can lead to misunderstandings about the etiology, signs and symptoms of diabetes and its management among people living in sub-Saharan Africa. These beliefs can influence the way individuals perceive and manage their diabetes. This finding was consistently supported by numerous studies. For example, many individuals in sub-Saharan Africa attribute the cause of diabetes to supernatural powers, punishment from gods or witchcraft (Ly et al., 2024; Omodara et al., 2022; Stephani et al., 2018). As a result, these superstitious beliefs about the cause of chronic diseases in sub-Saharan Africa make persons with diabetes reluctant to follow treatment regimens.

In sub-Saharan Africa, medical therapy for diabetes is scarce and expensive, causing many patients to turn to traditional therapies. Similarly, our review revealed that individuals with diabetes in sub-Saharan Africa frequently prefer traditional therapies. These therapies include spiritual healing or prayer sessions and traditional remedies, such as saltwater, bitter vegetables, and local plants, to neutralise and dilute blood glucose levels. However, it is still uncertain whether alternative medical practices can improve diabetes outcomes, and further investigation is needed. Nevertheless, systematic reviews have reported that traditional treatments and spiritual healing are common in sub-Saharan Africa, where plants such as Moringa oleifera, Cymbopogon citrullus, Hagenia abyssinica, Aloe vera, and Clausena are used specifically to treat type 2 diabetes (James et al., 2018; Love et al., 2022). Similarly, another systematic review highlighted that many people in Kenya, Cameroon, and Ghana use traditional healers and herbal remedies as part of their diabetes care plans because they believe that they can cure their condition (Stephani et al., 2018; Suglo & Evans, 2020). Other studies have also highlighted that bitter melon (Momordica charantia) and bitter foods, which have been used for centuries, are effective herbs for decreasing blood glucose levels (Chrystal, 2012; Gall et al., 2021; Mahwish et al., 2021; Omodara et al., 2022; Sari et al., 2022). Additionally, a study from sub-Saharan Africa revealed that plant-based diets, low in fat and high in fibre, are prevalent and beneficial for diabetes management (Steyn & Levitt, 2003).

Insulin therapy forms a cornerstone of the pharmacological management of diabetes; however, cultural beliefs and practices can significantly influence an individual’s understanding of diabetes and self-management, potentially leading to insulin refusal due to cultural values and a lack of knowledge about diabetes and the role of insulin therapy. Cultural beliefs in sub-Saharan Africa may cause suspicion or scepticism towards insulin due to a preference for traditional healing practices (Gill et al., 2009; Love et al., 2022). For example, in some sub-Sahara African cultures, insulin is viewed as an impairment that signals serious sickness and causes more complications in the long term (Mbanya & Ramiaya, 2006). This scepticism is not limited to sub-Saharan Africa; similar resistance to insulin is observed among African Americans, who may fear potential organ damage (Aikens & Piette, 2009). Additionally, people with diabetes may reject or alter recommended therapy if healthcare providers’ treatment plans are socially inappropriate (Carmel & Mishali, 2023; Raghavendran et al., 2020).

In exceptional instances, hyper religiosity, characterised by intense religious beliefs, or an unwavering dedication to rigid religious doctrines and practices coupled with avoidance behaviours, may adversely impact the effective management of diabetes. This might manifest as medication noncompliance and result in suboptimal control of blood glucose levels (Duke, 2021; Onyishi et al., 2022). For example, some Muslim patients are concerned about the source of their insulin, believing it is a porcine derivative, which goes against their religious beliefs and teachings, making them hesitant to use it, which can impact glycaemic control and result in complications (Caballero, 2018; Raghavendran et al., 2020; Rebolledo & Arellano, 2016).

Cultural beliefs and practices play crucial roles in shaping diabetes self-management behaviours, particularly with respect to diet and exercise. In many cultures, being overweight is seen as a symbol of wealth, beauty, or fertility, whereas losing weight is seen as a problem. Studies from Fiji, Nauru, Jamaica, Tonga, and many sub-Sahara African and Arabic countries report that larger bodies symbolise health and community connectedness, whereas those who lose weight or are thin are regarded with suspicion or pity (Caballero, 2018; Mora & Golden, 2017; Naigaga et al., 2018). Similarly, African American, and Latino or Hispanic women with type 2 diabetes may perceive being overweight as healthier than normal weight (Weitzman et al., 2013). A review in Nigeria revealed that physical activity can cause miscarriage in pregnant women, impacting diabetes management, as people may not engage in recommended physical activity (Chinenye & Ogbera, 2013). Such perceptions may lower exercise adherence among diabetic individuals (Nance et al., 2022). This cultural belief can impact dietary choices and hinder effective weight management.

Sub-Saharan Africa is home to both Christianity and Islam, with traditional African religious practices still prevalent. Our review highlighted that community organisations, religious communities and prayers are considered beneficial for losing weight and establishing good eating habits. According to a scoping review, Christian healing practices in Cameroon include special diabetes prayers, communion, oil anointing, and fasting, which help with diabetes management (Zimmermann et al., 2018). Additionally, Muslim prayers can regulate blood glucose, reduce cholesterol, alleviate anxiety, and improve overall physical health (Colberg et al., 2016; Ghazal, 2018). Similarly, a study among African Americans revealed that attending religious services at least once a week led to weight loss and less psychological distress than attending religious services less frequently (Nam, 2013; Yeary et al., 2020). Practising yoga and meditation has been shown to improve strength and blood glucose management (Ahmad et al., 2022). The probable reason might be the belief that taking care of one’s body as a temple and honouring God through healthy choices can be a driving force for weight loss, healthy eating habits, regular exercise, and long-term lifestyle changes all of which are rooted in principles of treating the body with respect and making choices that promote well-being (Chika Anekwe, 2021).

Cultural views on fruits and vegetables in sub-Saharan Africa can influence eating choices and self-management behaviours. Many sub-Saharan African cultures consider fruits and vegetables as food primarily for the poor, which affects their consumption and role in dietary management for conditions like type 2 diabetes. Local studies have shown that poor consumption of fruits and vegetables is prevalent among people with diabetes in the region, predisposing them to complications such as ulcers and exacerbating their condition (Rosane et al., 2024; Stephani et al., 2018; Zwane et al., 2023). These cultural beliefs and practices are not limited to sub-Saharan Africa. According to studies among South Asians and Afro-Caribbean women with type 2 diabetes in the UK, fruits and vegetables are believed to increase blood glucose levels, potentially causing diabetes (Chrystal, 2012; Patel et al., 2021), whereas Fijians believe that some fruits and vegetables are not healthy for children (Hawea et al., 2021). In another case, cultural beliefs among Javanese people attributed some fruits or vegetables with exceptional healing properties, encouraging individuals to prioritise consuming them in their diets (Sari et al., 2022).

Community organisations, and cultural, spiritual, and religious beliefs can significantly affect diabetes management in sub-Saharan Africa. Our review highlighted those practices and beliefs, such as fasting and spiritual healing, that can help people with diabetes cope with the emotional and psychological challenges of the disease, medication adherence, and better medical appointments. These practices and beliefs promote medication adherence, improve medical appointments, and provide support, encouragement, and hope during illness, thereby enhancing mental and physical well-being in diabetes management. These findings align with results observed in multiple studies conducted in Alabama (USA), Indonesia, and the Islamic Republic of Iran (Gore et al., 2012; Gugun et al., 2021; Javanmardifard et al., 2020). Similarly, religious coping helps PWD quit smoking, improve diet and exercise, reduce alcohol consumption, manage medications, and manage health (Duke, 2021; Sridhar, 2013). In black women with type 2 diabetes, praying and meditation are associated with improved glycaemic control and effective diabetes management (Darvyri et al., 2018; Onyishi et al., 2022; Weber & Doolittle, 2023). There is promising evidence that intermittent religious fasting could be an effective diabetes treatment. It has been shown to reduce body weight and lower levels of fasting glucose, insulin, adiponectin, and leptin (Ahmad et al., 2022; Albosta & Bakke, 2021). Additionally, it may even reverse insulin resistance, potentially reducing the risk of developing type 2 diabetes and improving overall glycaemic control (Albosta & Bakke, 2021; Saeed et al., 2021). Another study in Türkiye revealed that individuals with good medication use, diet, and exercise habits experienced higher levels of positive religious coping (Celik et al., 2022). Praying and following religious practices may positively impact self-management by providing inner peace and strength, whereas faith communities offer emotional and psychological support for diabetes management.

Diabetes stigma is a growing concern in sub-Saharan Africa, with individuals fearing stigmatisation and reluctance to share their condition with friends, family, and relatives. This finding aligns with a systematic review of studies that revealed that PWD in sub-Saharan Africa often faces stigma and confusion, which may lead to negative outcomes (Love et al., 2022; Zimmermann et al., 2018). Stigma disproportionately affects individuals with higher BMI and HbA1c or poorer blood glucose control, suggesting that those who need the most help are also the most affected by stigma (Liu et al., 2017).

Finally, our review highlighted that PWDs who have strong community organisations, social networks and support tend to have better functioning, fewer psychological issues, and improved diabetes self-management. This is supported by the findings of numerous studies (Chan et al., 2020; Karimy et al., 2018; Ramkisson et al., 2017), as community organisations such as religious communities and church members offer emotional encouragement and practical assistance, such as transportation and grocery shopping, to help individuals manage their condition more effectively (Darvyri et al., 2018; Onyishi et al., 2022; Sridhar, 2013; Watkins et al., 2013). The psychological impact on people with diabetes may be improved by increasing community support and acknowledging informal care.

## Limitations and Strengths of this Review

This review may be limited by the inclusion of papers regardless of quality, and the exclusion.

of non-peer-reviewed articles and studies published in languages other than English, which may have resulted in the omission of relevant research. Additionally, the nature of the studies retrieved predominantly qualitative and cross-sectional precludes the applicability of statistical analysis and hinders the ability to infer cause-and-effect relationships.

Moreover**,** the cultural, religious, or spiritual lens used by certain authors to interpret the results of the included studies may have influenced their subjective interpretation of the data, leading to biased reporting of outcomes.

Despite these limitations, this review possesses notable strengths. It represents the first integrative review that provides fresh perspectives on the impact of community organisation, culture, religion, and spirituality in the management of diabetes in sub-Saharan African countries. Furthermore, a diverse range of studies was included, most of which exhibited high methodological quality. As such, this review makes valuable contributions to the field of diabetes research by consolidating relevant evidence specific to the sub-Saharan African context.

## Implications for Practice

To improve diabetes self-management in sub-Saharan Africa, it is important to integrate cultural beliefs and social norms into diabetes education programs and healthcare services. This approach may help achieve more effective and culturally appropriate care for people with diabetes in the region. Providing culturally sensitive diabetes care is essential for addressing cultural beliefs and practices that can be barriers to accessing social support for people with diabetes in sub-Saharan Africa. This might be achieved by involving community leaders and traditional healers in diabetes education and care programs. Healthcare providers and diabetes educators could implement culturally sensitive and affordable diabetes self-management education and support families and community members to improve social support for people with diabetes in sub-Saharan Africa. While prayer- and faith-based interventions may be useful, they should not replace medical treatment or advice from healthcare professionals. Furthermore, clinicians could also ask patients about their religious or spiritual practices and their influence on diabetes-related self-management behaviours in clinical practice.

## Implications for Policies

Policymakers in sub-Saharan Africa must consider the significant influence of community organisations, cultural attitudes, social norms, traditional beliefs, and normative impacts on health behaviours. These factors shape how individuals perceive illness and interact with healthcare. Policies could integrate these cultural dimensions to create sustainable and culturally appropriate interventions.

Both the national and local levels could develop and implement policies that support community-based interventions for diabetes management. This could include funding for community health programs, training healthcare workers in cultural competency, and the development of guidelines for integrating traditional and biomedical practices. Community health workers (CHWs), closely embedded in local communities, can be trained to provide diabetes education and support. Health workers can act as intermediaries between the healthcare system and the community, offering culturally relevant advice and support that aligns with the community’s beliefs and practices.

Furthermore, it is crucial for governments to support and establish guidelines to oversee the activities of prayer camps. Providing basic training for religious leaders and traditional healers on diabetes, its management, and patient rights is essential. This approach can help bridge the gap between traditional and modern healthcare systems.

## Implications for Future Research

Further research is needed to fully understand how sub-Saharan African culture, religion, spirituality, and social support impact diabetic self-management. Therefore, more qualitative research is needed to better understand how culture and community organisations impact social support and self-management. There is some evidence suggesting that prayer and faith-based interventions could help improve glycaemic control in the management of diabetes. However, it is important to develop social support interventions that are culturally tailored to the sub-Saharan African context to address this issue effectively.

## Conclusion

This comprehensive literature review analysed 47 studies that investigate the roles of community organisations, religion, spirituality, and cultural beliefs in providing social support and facilitating self-management for diabetes in sub-Saharan Africa. The review encompassed a variety of research methodologies including qualitative, quantitative, mixed-methods, and non-randomised clinical trials, although many of the studies were qualitative in nature.

The understanding and management of diabetes in many sub-Saharan African societies are often clouded by misconceptions and cultural beliefs, which are frequently intertwined with religious and spiritual perspectives as well as significant life events. The sociocultural context of a region significantly influences individuals’ attitudes towards medical treatments, dietary choices, and levels of physical activity, ultimately affecting diabetes management.

Faith-based community organisations and religious institutions play a pivotal role in enhancing diabetes self-management within these communities. They provide vital social support that can empower individuals living with diabetes. These organisations promote healthy eating habits, often encouraging practices like fasting and prostration that resonate with cultural and religious norms. Such practices not only foster a sense of community and belonging but may also contribute positively to weight management and metabolic control.

Furthermore, the behaviours encouraged by these organisations can serve as effective coping mechanisms for individuals managing diabetes, potentially leading to improved glycemic levels. The literature review highlighted that robust community organisations and strong social support networks are crucial in enhancing individuals’ capacity for self-management. These social structures can assist communities across sub-Saharan Africa in achieving better glycemic control and overall health, thereby addressing the dual challenges of diabetes and its related complications.

## Appendix 1

See Table

**Table 3:** Characteristics and findings of the included studies that examined the role of community organisation, religion, spirituality and cultural beliefs in diabetes social support and self-management in sub-Saharan Africa

Authors/year	country	Purpose/aim	Design	Population/sample	key findings/outcome measures
(Abdulrehman et al., 2016)	Kenya	To describe diabetes self-management among the Swahili of coastal Kenya and explore factors that affect diabetes self-management within the context of Swahili culture	Ethnographic research.**	30 Swahili male and female participants aged 18 and older, with type 2 diabetes	Diabetes self-management is inadequate due to a limited understanding of the disease, local beliefs, family and religious contexts, cultural values, belief systems, and economic factors like poverty and high biomedical care costs also influence self-management behaviour. as well as the impact of sociocultural, educational, and social support all contributed to the challenges faced in managing diabetes
(Adejoh, 2014)	Nigeria	The study examined the association and influence of diabetes knowledge and health beliefs on diabetes management among the Igala, Nigeria	Cross-sectional survey.*	152 people with diabetes	A significant relationship was found between diabetes knowledge, health beliefs, and diabetes management. Perceptions of severity and benefits were shown to positively influence diabetes management. The regression analysis indicated that both knowledge and health beliefs are crucial factors for effective diabetes management
(Affusim & Francis, 2018)	Nigeria	This study attempted to ascertain how family/social support influences adherence to diabetic management	Cross-sectional survey.*	158 people with diabetes	The study found that social support significantly influenced various aspects of life, including medication adherence, clinic attendance, BMI, and blood glucose levels, and family members play a significant role in establishing a supportive environment and amending supportive behaviours, such as reminding patients and supervising healthy diets
(Agofure et al., 2018)	Nigeria	To assess the knowledge of dietary and medical management of T2DM in Delta State Nigeria	The qualitative study used an in-depth interview guide.**	20 people with T2 diabetes	The study revealed that some individuals believe diabetes causes are superstitious, leading to the use of herbs for treatment, often due to financial constraints and cultural influences, which negatively impact diabetes management
(Aikins, 2005)	Ghana	To outline approaches towards improving patient-centred health care and policy development regarding diabetes in Ghana	A longitudinal qualitative study with individual interviews, group interviews, and ethnographies.**	67 people with diabetes	The results of the research showed that participants preferred biological management, whereas cure-seeking and medical inactivity were motivated by the psychosocial effects of diabetes and spiritual and theological reasons for diabetes care. Diabetes was linked to bad living habits, spiritual disruption, witchcraft, and sorcery. Traditional and religious healing methods were sought, but eventually biomedical intervention was sought due to deteriorating physical health. Christian prayer and communion were accepted as spiritual mediation for diabetes treatment. They engaged in active cure-seeking, combining ethnomedical and faith healing systems to relieve drug and dietary management, improving their mental and physical well-being
(Alaofè et al., 2021)	Benin	To explore specific cultural beliefs, attitudes, behaviours, and environmental factors to help adapt a diabetes self-management program to patients with T2D from Cotonou, in southern Benin	The qualitative study used Focused Group discussion.**	32 T2D patients, 12 Community and 16 academic partners of those who lived in Cotonou for at least six months each year	Along with challenges of food access, cultural perception and norms also have an impact on self-management, particularly regarding fruits and vegetables because they have historically been associated with the underprivileged as commodities as well as the information about local food selections and recipes. Participation and engagement in physical activities were promoted by group support and neighbourhood factors. Religion, on the other hand, was used as a coping method to ward off unpleasant emotions. However, negative perceptions and social stigma hindered healthy coping with stress
(Ameyaw Korsah & Ameyaw Domfeh, 2020)	Ghana	The study explored the realities of religiosity/spirituality in coping among people with diabetes and implications for policy formulation in Ghana	The hermeneutic phenomenological Research approach used individual interviews.**	37 patients with both type 1 and 2 diabetes who attended the Outpatient department regularly	The study examined the spiritual experiences of patients with diabetes mellitus. It looked into their beliefs in God as a source of hope, their use of prayers, invocations, and fasting, and their conviction that God can heal diabetes through other people (doctors, and nurses) and the effectiveness of prayer as a coping mechanism and spiritual support for lowering blood glucose levels. The study also explored issues such as abuse in spiritual prayer camps accused of causing their condition through bad demeanours and sinful behaviours. In another way, they were asked to pay a special fee for the pastor to fast and pray for healing which leads to economic drainage in addition to the diabetes burden
(Assah et al., 2015)	Cameron	To examine the effectiveness of a community-based multilevel peer support intervention in addition to usual diabetes care on improving glycaemic levels, blood pressure and lipids in patients with Type 2 diabetes in Yaounde, Cameroon	Nonrandomised controlled trial.#	People with T2 diabetes (96 subjects in the intervention arm and 96 age and sex-matched controls were recruited into the control arm)	The intervention group experienced a significant reduction in HbA1c, cholesterol, HDL, BMI, and diastolic pressure over 6 months. Peer support also substantially reduced fasting blood glucose, cholesterol, HDL, BMI, and diastolic pressure. Additionally, diabetes self-management behaviours in the intervention group improved significantly over the 6 months
(Atinga et al., 2018)	Ghana	To explore how and why chronic diabetic and hypertensive patients admitted to long-term drug therapy poorly adhered to prescribed medication taking	The qualitative study used in-depth interviews. **	32 patients admitted to long-term diabetes and hypertension drug therapy in the hospital and 17 caregivers	The study reveals that nonadherence is influenced by patients who often believe their illness is caused by curses, witchcraft, and family spells, leading them to switch to spiritual healing methods like prayers, fasting, and paranormal approaches. These methods can influence suboptimal self-management, discourage medication use, and negatively impact patient outcomes. Cultural and social norms also influence medication discontinuation, with some patients discouraged by societal norms and others believing certain morbidities, like diabetes, are trivialised. Factors such as peer pressure, family, affordability, and herbal remedies also contribute to this issue
(Awah et al., 2008)	Cameroon	To explore the cultural aspect of compliance, its underlying principles and how these cultural aspects can be used to improve patient-centred care for diabetes in Cameroon	Ethnography qualitative study (comparative anthropological approach). **	20 people with T2 diabetes	The ethnography revealed a lack of knowledge about diabetes and risk factors among individuals with the condition. Patients with diabetes believe their problems are only partially understood by biomedical healthcare providers, leading them to resort to traditional medicine or rejection or modification of treatment. They adopt other treatments in combination with biomedicine, believing they will be more effective in curing diabetes which will lead to less efficacy. Biomedical therapy compliance faced setbacks due to competing treatment regimes, diabetes treatment costs, and AIDS’ impact on weight loss. People with diabetes had negative opinions about certain prescriptions, fearing they could interfere with their social image and ancestry, leading to concerns about bridging ancestry relationships and weight loss during the HIV/AIDS era
(Baumann et al., 2015)	Uganda	To test the feasibility of a peer support intervention by a nurse-led interdisciplinary team	Quasi-experimental study. #	46 people with T2 diabetes (19 champions and 27 partners were recruited)	The study found no differences in pre- and postintervention measures between champions and partners. Healthy eating showed a positive change in health behaviours, while social support, emotional well-being, BMI values, and self-management confidence remained unchanged. Average diastolic blood pressure and A1C values decreased. However, Peer intervention improved diabetes care, increased patient knowledge, and improved energy and pain, focusing on healthy eating, medication, exercise, and emotional well-being
(Bayked et al., 2022)	Ethiopia	To examine type 2 diabetes patients’ perceptions of the causes of their illness in Northeast Ethiopia	The qualitative study used in-depth interviews. **	24 people with T2 diabetes	The study findings revealed that patients were linking the cause of their diabetes symptoms to specific traumatic life events, like emotional upheavals, financial difficulties, and spiritual influences. This mindset had a detrimental effect on their ability to manage their diabetes effectively
(BeLue et al., 2018)	Senegal	To identify beliefs, the role of culture, context, and experiences related to diabetes management in a diverse sample of Senegalese families	Exploratory qualitative study. **	20 people living with T2 diabetes and 19 caregivers (Family members)	People with diabetes face physical, emotional, and financial stressors while managing their condition. Family caregivers provide instrumental, emotional, financial aid, and informational support, assisting with doctor appointments, diet management, medication management, mental and emotional support, and other miscellaneous tasks, however, Caregivers face stress, reduced quality of life, and fatigue from caregiving tasks related to diabetes
(Belue et al., 2012)	Senegal	To examine experiences with diabetes self-management among clinic patients residing in M’bour, Senegal, using the PEN3 model as a cultural framework	The qualitative study used narrative interviews. **	54 people with diabetes	In this study, Family plays a crucial role in managing diabetes by providing support and care, even with limited resources. Participants identified both positive and negative contributions of family and friends to diabetes management. All the study participants were more relying on adult children, spouses, and community members for financial support. However, competing obligations of family members and friends leave some women with little support. Women often struggle to find support from their neighbours, who may not provide enough for their needs and mentioned how diabetes affects their relationships with family members. This is not surprising given that most daily activities related to diabetes management, including eating, interpersonal interactions, and financial decisions, are all decided on the family system level. Diabetes management faces barriers due to high modern medicine costs, leading to traditional plant-based medicine due to sociocultural influences and affordability
(Berkoh et al., 2022)	Ghana	The present study examines factors related to the noncompliance behaviour of patients receiving treatment at a diabetes clinic in the rapidly urbanising Bono Region of Ghana	A mixed approach using individual interviews. ^M^	165 people with diabetes	The majority of participants reported optimum compliance with diabetes medication, with some reporting suboptimum compliance (24.4%). Misconceptions about religious beliefs, psychosocial factors like stress, inevitable fate, herbal preference, fear of side effects, fatigue, health-seeking behaviour, and socioeconomic status contribute to suboptimum compliance
(Botchway et al., 2022a, 2022b)	Ghana	To evaluate how the frequency of participation in religious activities and the frequency of seeking care from TM practitioners influence HbA1c control among Ghanaian adults with T2DM	Cross-sectional survey. *	254 people with T2 diabetes	The study indicates that increased participation in religious activities correlates with reduced HbA1c levels. Conversely, it was found that greater use of Traditional Medicine practitioners was significantly linked to elevated HbA1c levels
(Botchway et al., 2021)	Ghana	To apply a social network approach in examining how diabetes-related stigma may influence perceived social support and glycaemic control among Ghanaian adults with T2DM	Cross-sectional survey. *	254 people with T2 diabetes	Kin composition and household composition significantly impact social support, adjusting for self-stigma. Self-stigma moderates the relationship, but not household or network density. Low self-stigma participants show a significant association, while network size positively influences social support. Diabetes-related stigma does not moderate associations, and social support does not significantly affect glycaemic control
(Botchway et al., 2022a, 2022b)	Ghana	To examine the effects of compositional, structural, and functional dimensions of social networks on HbA1c control among adults with T2DM in urban Ghana	A hospital-based, cross-sectional survey. *	254 People with T2 DM	The study revealed that although gender and social support did not have a significant impact on each other, having a larger family and living with more people were strongly associated with increased social support. However, there was no significant relationship found between network characteristics and social support with HbA1c
(de-Graft Aikins et al., 2015)	Ghana	To examine explanatory models of diabetes and diabetes complications among urban poor Ghanaians living with diabetes and implications for developing secondary prevention strategies	Qualitative study using an individual interview guide. **	20 people with T2 diabetes	The study found that the misconceptions and misunderstandings of the causes of diabetes and its complications are linked with beliefs, culturally held theories, supernatural causes, social contexts, and life events. Diabetes management is influenced by traditional and cultural models, with subjective experience and poverty playing significant roles in suboptimal self-management practices. Cultural representations of the paranormal impact people’s perception of their social environment and healthcare-seeking, potentially counteracting the beneficial effects of biological treatment
(Doglikuu et al., 2021)	Ghana	Aim to investigate how household socioeconomic status and neighbourhood support system are associated with adherence to dietary recommendations among persons with T2DM in Ghana	Facility-based cross-sectional-survey. *	530 persons living with T2 DM	Age, marriage, moderate social support, and high socioeconomic status were found to be significant factors affecting dietary recommendation adherence. Marriage was the most significant, while moderate social support and low social support were less significant. High socioeconomic status was also associated with dietary recommendation adherence
(Foley & BeLue, 2017)	Senegal	To identify potential cultural enablers and barriers to dietary management of type 2 diabetes in the urban setting of M’Bour, Senegal	Qualitative study. **	41 people with T2 diabetes	The study found that family support, multiple diabetics in the household, and access to healthy food positively influence diabetes management. However, barriers like a lack of understanding of the causes of diabetes, financial challenges, social isolation, abandonment, cultural or traditional diets, and conflict between local and diabetes-appropriate foods hinder self-management among people with diabetes
(Habte et al., 2017b)	Ethiopia	To explore medication-related perceptions of adult patients with type 2 diabetes attending treatment in public hospitals of urban centres in central Ethiopia	The qualitative study used individual interviews. **	39 people with T2 diabetes	The study found that local sociocultural contexts impact medication-related perceptions among participants, emphasising the need for anti-diabetic medications. Patients often have concerns about adverse effects, inconveniences, and access, which can negatively affect adherence and potentially impact health outcomes. Insulin is associated with higher severity due to patients believing it was prescribed when their diabetes was not responding to other treatments. Adverse effects, such as falls, paralysis, and weight gain, are linked to insulin. The influence of others on insulin initiation, along with negative aspects like scars and inability to discontinue it during holy water and thought to further complicate the situation
(Habte et al., 2017a)	Ethiopia	To conduct an exploratory study using qualitative methods to elicit the barriers and facilitators to adherence to recommended anti-diabetic regimens of patients with type 2 diabetes	Qualitative research design with in-depth interviews. **	39 people with T2 diabetes	Diabetes self-management was impacted by a variety of factors, such as patient self-efficacy, social support from family and close friends, medication-related concerns, religious beliefs (such as holy water use and fasting, refusal of insulin due to religious reasons), healthcare providers’ perceptions of the healthcare system, and illness perceptions (such as lack of acceptance, asymptomatic status, limited knowledge, or chance diagnosis and hope for a cure)
(Hjelm & Mufunda, 2010)	Zimbabwe	To explore beliefs about health and illness that might affect self-management and health-care-seeking behaviour in persons diagnosed with diabetes mellitus living in Zimbabwe	The exploratory study used individual interviews. **	21 people with diabetes	The study revealed that diabetes is believed to be caused by factors such as supernatural influences like fate, punishment from God, and witchcraft. As a result, they try to improve health, suggested by prayers, healing powers, and household remedies. Family members provided material support, including money for food, drugs, and self-monitoring equipment. Respondents mainly followed the advice, focusing on diet, medication, check-ups, and stress management. However, limited knowledge about diabetes and Self-management is limited, but a combination of individual measures, household remedies, and religious acts is often used
(Jaafaripooyan et al., 2021)	Ethiopia	To determine the social support magnitude, types, and roles in diabetes management among people with diabetes in southern Ethiopia	Facilities-based cross-sectional study. *	634 patients with diabetes	The finding stated that half of the diabetic patients receive their social support from families, close relatives, or friends, with nonmaterial support being more prevalent than instrumental support. The study found that social support plays a significant role in diabetes management, with the odds of receiving support increasing by 10% as educational attainment advanced. Factors such as place of residence, occupation, presence of diabetic families, nondiabetic acute medical conditions, blood glucose levels, medical follow-up compliance, and taking anti-diabetic drugs a day before the current visit to a healthcare facility were all statistically significantly associated with social support
(Korsah, 2023)	Ghana	To explore the use of religious capital as a coping strategy by individuals with type 2 diabetes mellitus in self-management at the Techiman Holy Family Hospital Diabetes Clinic in the Bono East Region of Ghana	The exploratory qualitative research design used individual interviews. **	27 people with type 2 diabetes mellitus with no experiences of comorbidities	The study found that religious capital as a coping strategy for patients with type 2 diabetes consists of five core areas: (1) Prayer and Fasting for Courage from God, (2) Reliance on God as the Creator of Human Beings who Cures and Heals Diseases in the Body, (3) God as the Source of Life in times of Illness, (4) Faith and Hope in God, and (5) Doctors and Nurses as Surrogates for God, which emphasises the importance of faith and hope in managing diabetes mellitus. These core areas help patients cope with diabetes-related distress and challenges following diagnosis
(Lloyd et al., 2023)	Kenya	To draw on the findings of this research to develop educational materials addressing the stigma surrounding diabetes and depression	The qualitative study/phenomenological approach used individual interviews and Focused group discussion. **	with health care providers, people with comorbid diabetes and depression, community members/A total of six focus group discussions (6–8 participants per group) and 10 key informants	The study highlights that cultural misconceptions about the Symptoms of diabetes were often rooted in communities’ social, behavioural, and cultural contexts and inappropriate language use leading to isolation from the community and stigma, which affects glycaemic control and diabetes self-management. Community and religious leaders promoted open interaction, spiritual guidance, and support, while herbalists and doctors were seen as alternative treatments. Informal careers provide support to people with diabetes, reducing anxiety and emotional support. Enhancing community support and recognising informal care improves the psychological impact of people with diabetes
(Lugaya et al., 2022)	Kenya	To determine the level of social support received by Diabetes Mellitus Type II patients attending a County Referral Hospital in Kenya	The descriptive cross-sectional study design. *	168 people with T2 diabetes	A study found that many people with Type II Diabetes do not have enough social support, which can lead to suboptimum self-management habits due to a lack of support from family and peers. The study also highlights the strong connection between proper self-management and social support for those with Type II Diabetes Mellitus
(Masupe et al., 2022)	South Africa	To unravel experiences, identify self-management barriers, and solicit solutions for enhancing self-management of T2D/HTN from patients and their healthcare providers	The qualitative study used individual interviews. **	43 people with T2DM/HTN 3 clinical nurse practitioners (CPNs), 1 dietician, and 1 health	The finding shows that self-management in real life is complex, with patients having to deal with cultural norms that prevent lifestyle modification, bad relationships, postdiagnosis grief, and communication gaps that cause social stigma, entitlement mentality, and insufficient community services, all of which contributed to less-than-ideal self-management
(Masupe et al., 2018)	South Africa	To evaluate the outcomes of the self-management approach and added benefit of the community component compared to the facility component only; and to translate the findings in dialogue with stakeholders into relevant input for national guidelines and policies, using reciprocal knowledge transfer across the different sites	A qualitative study with a phenomenological design using in-depth interviews and focus group discussions (FGDs). **	Adult patients with T2DM, health care workers involved in the care and management of T2DM patients including physicians, nurses, and community health workers in charge of the health clubs	The study findings stated that DM is a complex disease influenced by sociocultural factors, affecting patients’ physical, social, and emotional effects. Factors influencing diabetes self-management identified include cultural beliefs, cultural practices, and cost of treatment. Participants also reported personal discipline, religious practices, peer pressure, healthcare professional attributes, fear of diagnosis, and convenient ignorance all contributed to suboptimal diabetes self-management
(Mekashaw Bayked et al., 2022),	Ethiopia	The study explored the experiences of type 2 diabetes patients in Northeast Ethiopia regarding folk healing alternatives	The qualitative study used individual interviews. **	24 people with T2 DM	The finding revealed that Patients viewed allopathic alternatives to folk healing positively, believing they were deceivers. They used folk medicines to address diabetes’ consequences, with spiritual and herbal healing being the most common approaches
(Mogre et al., 2019)	Ghana	To explore patient and healthcare provider (HCP) perspectives about patients’ barriers to the performance of diabetic self‐care behaviours in Ghana	Qualitative (Semi‐structured in‐depth individual interviews). **	23 people with type 2 diabetes patients and 14 HCPs (healthcare providers)	People with diabetes often have misconceptions about diabetes and its care, this misperception can hinder diabetes self-management and hinder adherence to dietary recommendations. This leads to seeking care from alternative sources like prayer camps, herbalists, local medications, and spiritual healings instead of visiting the hospital. Insufficient family support can also hinder diabetes self-management. Culture influences dietary advice adherence, affecting food choices and it is believed that being fat and plumpy is a sign of good living and wealth and losing weight is a sign of having a problem. Culturally, others perceived that exercise is for the rich or it is a Western culture and hence will not participate in it if they think they are poor. Patients fear stigmatisation due to their condition, leading to reluctance to share it with friends, family, and close relatives. This affects self-management behaviours and follow-up at clinics
(Mphasha et al., 2022)	South Africa	To explore family support in diabetes management	A qualitative study (semi-structured interviews. **	17 people with diabetes	Family members provided support for self-management activities postdiagnosis with diabetes, including medication intake, insulin injections, lifestyle modifications, healthy food consumption, food preparation, and physical activity. Diabetic men with sexual dysfunction also reported getting similar support from their wives whereas on the contrary, diabetic women do not get sufficient support from their husbands
(Mphwanthe et al., 2021)	Malawi	To qualitatively assess patient/client barriers and facilitators to achieving a healthy diet and physical activity to support T2DM self-management in adult Malawians	The qualitative study used individual interviews. **	39 people diagnosed with T2 DM	The study’s findings supported the existence of social support networks such as friends and family members play a crucial role in diabetes self-management. Social support and participation in diabetes peer groups are essential for maintaining healthy eating behaviours. However, Cost and access to food, difficulties with separate food preparation and purchase, attending events, and inconsistent dietary advice from many sources were some of the dietary barriers to self-management of T2DM. Coworkers and religious groups also provide support, while religious groups and prayer are believed to be essential for weight loss and healthy eating behaviours. Comorbidities and fear of public ridicule were key perceived barriers to participants being physically active
(Olagbemide et al., 2021)	Nigeria	To assess the relationship between family support and medication adherence among adult Type 2 DM (T2DM) attending the family medicine clinic of a rural tertiary hospital	Analytic cross-sectional survey. *	367 people with T2 diabetes	The study found that respondents with strong family support had medium/high adherence to their medications, with a higher proportion having good glycemic control. Strong family support also had a significant relationship with adherence level and glycemic control. The higher the level of adherence, the lower the mean FPG. Post hoc analysis showed significant differences between groups. Overall, strong family support was found to be a significant factor in promoting glycaemic control and maintaining good glycaemic control
(Olorunfemi & Ojewole, 2018)	Nigeria	To correlate medication belief, with medication adherence among patients with DM	Correlational research design. *	180 people with diabetes	The finding shows that people with diabetes had a negative perception of medication, believing it could cause harm or poison to their system, which negatively affects diabetes self-management. A statistical relationship was found between medication belief and adherence, with a p-value of 0.005
(Onyango et al., 2022)	Uganda	To extend the existing evidence to Uganda´s setting by determining the association between perceived social support from family and diabetes self-management behaviour among patients in the eastern region of Uganda	Quantitative cross-sectional survey. *	405 people with diabetes	The study revealed that participants received help at home to manage their diabetes, and more than half reported an intense sense of togetherness with their families. The study also found that family unity and financial assistance for medical expenses were intricately linked to better self-management practices
(Osuji et al., 2018)	Nigeria	Aimed to investigate the relationship between glycaemic control and perceived family support among Nigerians with type 2 diabetes mellitus	A quantitative cross-sectional survey. *	316 people with T2 diabetes	The study finds that 43.8% of T2DM patients had strong perceived family support, which was the independent predictor of glycaemic control
(Pienaar & Reid, 2021)	South Africa	To explore the experiences of adults with type 2 diabetes who took part in a diabetes peer support intervention that used the Mmogo method	The qualitative study used individual interviews. **	12 people with T2 diabetes	The Diabetes Peer Support Intervention resulted in positive lifestyle changes through regular conversations on diabetes-related topics. Participants gained a better understanding of diabetes, physical changes, and symptoms. This information enabled informed decisions, diet adjustments, and improved activity levels. The continuous support and interaction led to positive lifestyle changes, and improved confidence in self-management, clinical outcomes, and connectedness. Cultural perceptions of obesity and overweight influenced self-management
(Rotheram-Borus et al., 2012)	South Africa	To test the feasibility and acceptability of a mobile phone-based peer support intervention among women in resource-poor settings to self-manage their diabetes	Pilot study. *	22 Women with diabetes	The majority of women were attempting to understand "WHY" they got diabetes. Witchcraft and beliefs in retribution for transgressions were widespread. Women claimed to be quite religious. All of them prayed frequently, had daily spiritual experiences, and relied on God. BMI and blood glucose levels increased and decreased between baseline and three months, respectively. Social support and positive action coping styles increased and decreased, respectively. Spiritual hope decreased and diastolic blood pressure increased between three and six months. Emotional distress, systolic blood pressure, exercise, daily steps, and depression coping styles did not change significantly over time
(Shilubane et al., 2016)	South Africa	To explore and describe the beliefs and management practices of patients with DM	Quantitative cross-sectional survey. *	100 people with diabetes aged 40 years and above	24.0% of respondents believed diabetes can be cured, while 22.0% did not believe diet helps manage diabetes. 44.0% did not know if physical exercise could benefit patients, their beliefs affecting their self-management and potentially resulting in suboptimal self-management
(Tabong et al., 2018)	Ghana	To increase the understanding of the multifaceted psychosocial and cultural contexts of risk perception, premorbid behaviour, illness experience, management, adherence, and social attitudes to diabetes	Descriptive phenomenology uses individual interviews and Focused group discussion. **	73 people with diabetes	The finding shows that social support systems, such as networking with friends, relatives, religious bodies, and social clubs; and relying on their religious values, were beneficial coping mechanisms for diabetic patients. These networks provided emotional, encouraged medication intake, and financial support, as well as opportunities for various activities that can help alleviate concerns related to the disease
(Tusubira et al., 2021)	Uganda	To examine how patients engaged in self-management for diabetes and hypertension in rural Uganda	Cross-sectional qualitative used individual interviews. **	19 people who lived with diabetes and/or hypertension	The study findings clearly showed that patients prefer traditional medicine over conventional treatments such as saltwater, bitter vegetables, and local plants. In addition, individuals with diabetes often turn to their children for emotional and practical support while seeking social support to manage their condition. Community organisations provide a platform for mutual support, where individuals can obtain peer support and helpful assistance. Such support groups offer emotional and monetary assistance, help promote self-management, provide food assistance, and encourage regular appointment attendance
(Visagie et al., 2018)	South Africa	To discover and capture the quintessence of the social support experiences of a group of emerging adults with well-controlled type 1 diabetes; and how this contributed to their diabetes management	Descriptive qualitative research uses individual interviews. **	8 emerging adults with well-controlled T1DM comprised three males and five females	The finding indicates that receiving assistance from family members helps individuals feel more connected and empowered. This sense of belonging is particularly important for those with diabetes, as ongoing support can lead to positive outcomes. By receiving assistance, individuals are better able to understand the specific requirements of managing diabetes and promoting healthy self-management behaviours. Additionally, having friends involved in daily activities can provide support, such as monitoring blood glucose levels and offering helpful assistance. Building strong friendships can create a supportive environment where dietary needs are considered, leading to lower HbA1c readings. Taking initiative and being proactive can also positively impact those around them
(Werfalli et al., 2020)	South Africa	To determine the relationship of social support, especially that of family and friends with their self-management	Cross-sectional survey. *	406 people with T2 diabetes	Social support positively relates to self-management practice for diabetic meal planning, foot care, physical activity, and handling participants’ feelings about being diabetic and testing blood glucose. However, it does not affect medication use. Emotional support from family and friends positively impacts knowledge scores. Older age negatively impacts knowledge, while higher income positively impacts self-management practice. No significant associations were found between sociodemographic variables, HbA1c, and social support with knowledge or self-management practice
(Westaway et al., 2005)	South Africa	To determine the underlying dimensions of a social support measure and investigate the effects of social support on health, well-being, and management of diabetes mellitus (metabolic control and blood pressure (BP) control)	Cross-sectional, survey. *	263 people with Both T1&T2 DM	The study found no correlation between social support dimensions and metabolic control. Patients with controlled blood pressure had more socio-emotional, tangible, and overall support than those with poorly controlled blood pressure, suggesting social support is beneficial for managing diabetes
(Willemse et al., 2018)	South Africa	To characterise diabetes management experiences of South African young adults living with well-controlled type 1 diabetes	Exploratory qualitative used individual interviews. **	8 young adults with T1 diabetes	Young adults with diabetes experience better adjustment and contentment, relying on family support, friends, and medical staff for guidance and support in exploring meaning-making processes for effective glycaemic control

(* = QN studies; ** = QL studies; # = Non-RCT; M = Mixed)

3

## Appendix 2 Literature Search Terms Used to Conduct the Integrative Review

**Table:**

Concepts	Searching terms
Community organisations	(MH "Organi?ations, Nonprofit + ") OR (MH "Faith-Based Organi ?ations + ") OR (MH "Community Participation + ") OR "Community organi?ation*" OR (MH "Community Networks") OR (MH "Community Support") OR (MH "Community Integration") OR (MH "Community Resources + ") OR (MH "Organizations + ") OR "Religious Organi?ation*" OR "Community support*" OR "Community institution*" OR "community non-profit*" OR "Community association*" OR "Community Role*" OR "community group*" OR "community service*" OR "community program*" OR "social participation*" OR "welfare organi?ation*" OR "support group*" OR "Rehabilitation*" OR "Community-Based*"
	OR
Religion	(MH Religion +) OR "Religious Belief*" OR faith* OR Belief* OR "religious coping*" OR "pastoral care*" OR "Religious coping strateg*" OR "Religious Practice*" OR Prayer* OR Meditation*
	OR
Spirituality	(MH "Spirituality") OR (MH "Spiritual Therapies + ") OR (MH "Religion and Medicine + ") OR (MH "Traditional Medicine Practitioners") OR "Spiritual Care*" OR "Spiritual Support*" OR "Pastoral Care*" OR "Spiritual healing*" OR "spiritual coping*" OR "Spiritual Sensitivit*"
	OR
Cultural beliefs	"Cultural belief*" OR (MH "Culture + ") OR (MH "Cultural Competency") OR (MH "Social Norms + ") OR (MH "Social Factor*") OR (MH Ethnolog*) OR (MH "Community Support") OR "Health Belief*" OR "socio‑cultural*" OR "Cultural value*" OR "traditional belief*" OR "cultural practice*" OR "social identit*" OR "cultural sensitivit*"
	OR
Social support	(MH "Social Support + ") OR (MH "Psychosocial Support Systems") OR (MH "Family Support") OR (MH "Community Support") OR (MH "Social Work + ") OR (MH "Social Participation") OR (MH "Social Networking + ") OR "peer support*" OR "friend support*" OR "social relationship*" OR “perceived social support*" OR "perceived family support*" OR "received social support*" OR "group-based care*" OR "emotional support*" OR "instrumental support*" OR "appraisal support*" OR "romantic support" OR "significant other support*" OR "religious support*" OR "caregiver support*" OR assistance* OR "support group*" OR "support system*" OR "Social Support System*" OR "Psychosocial Support*" OR "Psychological Support System*" OR "Psychological Support*" OR "Decision-Making Support*"
	AND
Self-management	(MH "Self-Care + ") OR (MH "Self-Management") OR (MH "Blood Glucose Self-Monitoring") OR "Self-management*" OR "Disease management*" OR "Diabetes management*" OR "managing diabetes*" OR "self-management support*" OR "self-management behav*" OR "self-monitoring*" OR "physical activit*" OR "physical exercise*" OR diet* OR medication* OR "foot care*"
	AND
Diabetes	(MH "Diabetes Mellitus + ") OR (MH "Diabetes Mellitus, Type 2 + ") OR (MH "Diabetes Mellitus, Type 1 + ") OR Diabetes* OR "type 2 diabetes*" OR "type 1 diabetes*" OR "Diabetic Patient*" OR "people with diabete*" OR "patient with diabetes*" OR "Insulin-dependent diabetes mellitus*" OR "Non-insulin-dependent diabetes mellitus*"
	AND
Sub-Saharan Africa	(MH "Africa South of the Sahara + ") OR (MH "Sub-Saharan African People") OR (MH Africa +) OR (MH "Africa, Western + ") OR (MH "Africa, Eastern + ") OR (MH "Africa, Southern + ") OR (MH "Africa, Central + ") OR Angola* OR Benin* OR Botswana* OR "Burkina Faso*" OR Burundi* OR "Cabo Verde*" OR Cameroon* OR "Central African Republic*" OR Chad* OR Comoros* OR "Democratic Republic of the Congo*" OR "Republic of the Congo*" OR "Cote D’ivoire*"OR "Equatorial Guinea*" OR Eritrea* OR Eswatini* OR Ethiopia* OR Gabon* OR Gambia* OR Ghana* OR Guinea* OR "Guinea-Bissau*" OR Kenya* OR Lesotho* OR Liberia* OR Madagascar* OR Malawi* OR Mali* OR Mauritania* OR Mauritius* OR Mozambique* OR Namibia* OR Niger* OR Nigeria* OR Rwanda* OR "Sao Tome and Principe*" OR Senegal* OR Seychelles* OR "Sierra Leone*" OR Somalia* OR "South Africa*" OR "South Sudan*" OR Sudan* OR Tanzania* OR Togo* OR Uganda* OR Zambia* OR Zimbabwe*

## Appendix 3: Quality Appraisal Checklist

JBI critical appraisal checklist for qualitative research.

**Table:**

Authors	Q1	Q2	Q3	Q4	Q5	Q6	Q7	Q8	Q9	Q10	Score
(Abdulrehman et al., 2016)	Y	Y	Y	Y	Y	Y	Y	Y	Y	Y	10/10
(Agofure et al., 2018)	NC	NC	Y	Y	Y	NC	NC	Y	Y	Y	6/10
(Aikins, 2005)	Y	Y	Y	Y	Y	Y	Y	Y	Y	Y	10/10
(Alaofè et al., 2021)	N	NC	Y	Y	Y	NC	NC	Y	Y	Y	6/10
(Ameyaw Korsah & Ameyaw Domfeh, 2020)	Y	Y	Y	Y	Y	Y	Y	Y	Y	Y	10/10
(Atinga et al., 2018)	NC	NC	Y	Y	Y	NC	NC	Y	Y	Y	6/10
(Awah et al., 2008)	Y	Y	Y	Y	Y	Y	NC	Y	Y	Y	9/10
(Bayked et al., 2022)	Y	Y	Y	Y	Y	Y	Y	Y	Y	Y	10/10
(BeLue et al., 2018)	Y	Y	Y	Y	Y	NC	NC	Y	N	Y	7/10
(Belue et al., 2012)	Y	Y	Y	Y	Y	NC	NC	Y	Y	Y	8/10
(de-Graft Aikins et al., 2015)	Y	Y	Y	Y	Y	NC	Y	Y	Y	Y	9/10
(Foley & BeLue, 2017)	NC	NC	Y	Y	Y	NC	NC	Y	Y	Y	6/10
(Habte et al., 2017a)	Y	Y	Y	Y	Y	N	N	Y	Y	Y	8/10
(Habte et al., 2017b)	NC	Y	Y	Y	Y	NC	NC	Y	Y	Y	7/10
(Hjelm & Mufunda, 2010)	Y	Y	Y	Y	Y	NC	NC	Y	Y	Y	8/10
(Korsah, 2023)	Y	Y	Y	Y	Y	NC	NC	Y	Y	Y	8/10
(Lloyd et al., 2023)	Y	Y	Y	Y	Y	NC	NC	Y	Y	Y	8/10
(Masupe et al., 2022)	y	y	y	y	y	y	y	y	y	y	10/10
(Masupe et al., 2018)	Y	Y	Y	Y	Y	Y	NC	Y	N	Y	8/10
(Mekashaw Bayked et al., 2022)	NC	Y	Y	Y	Y	Y	Y	Y	Y	Y	9/10
(Mogre et al., 2019)	Y	Y	Y	Y	Y	NC	N	Y	Y	Y	8/10
(Mphasha et al., 2022)	Y	Y	Y	Y	Y	Y	Y	Y	Y	Y	10/10
(Mphwanthe et al., 2021)	NC	Y	Y	Y	Y	NC	NC	Y	Y	Y	7/10
(Pienaar & Reid, 2021)	Y	Y	Y	Y	Y	Y	Y	Y	Y	Y	10/10
(Tusubira et al., 2021)	NC	Y	Y	Y	Y	NC	NC	Y	Y	Y	7/10
(Tabong et al., 2018)	Y	Y	Y	Y	Y	Y	Y	Y	Y	Y	10/10
(Visagie et al., 2018)	Y	Y	Y	Y	Y	NC	NC	Y	Y	Y	8/10
(Willemse et al., 2018)	Y	Y	NC	Y	Y	NC	NC	Y	Y	Y	7/10

JBI critical appraisal checklist for analytical cross-sectional studies									
Authors	Q1	Q2	Q3	Q4	Q5	Q6	Q7	Q8	Score
(Adejoh, 2014)	Y	Y	Y	Y	NC	NC	Y	Y	6/8
(Affusim & Francis, 2018)	Y	Y	NC	Y	NC	NC	Y	Y	5/8
(Botchway et al., 2021)	Y	Y	Y	Y	Y	Y	Y	Y	8/8
(Botchway et al., 2022a, 2022b)	Y	Y	Y	Y	Y	Y	Y	Y	8/8
(Botchway et al., 2022a, 2022b)	Y	Y	Y	Y	Y	Y	Y	Y	8/8
(Doglikuu et al., 2021)	Y	Y	Y	Y	Y	Y	Y	Y	8/8
(Jaafaripooyan et al., 2021)	Y	Y	NC	Y	Y	Y	Y	Y	7/8
(Lugaya et al., 2022)	Y	Y	Y	Y	NC	NC	NC	Y	5/8
(Olagbemide et al., 2021)	Y	Y	Y	Y	Y	Y	Y	Y	8/8
(Onyango et al., 2022)	y	Y	NC	Y	NC	NC	Y	Y	5/8
(Olorunfemi & Ojewole, 2018)	Y	Y	Y	Y	NC	NC	NC	Y	5/8
(Osuji et al., 2018)	Y	Y	Y	Y	Y	Y	Y	Y	8/8
(Rotheram-Borus et al., 2012)	Y	Y	Y	Y	NC	NC	Y	Y	6/8
(Shilubane et al., 2016)	NC	Y	Y	Y	NC	NC	Y	Y	5/8
(Werfalli et al., 2020)	Y	Y	Y	Y	Y	Y	Y	Y	8/8
(Westaway et al., 2005)	Y	Y	Y	Y	Y	Y	Y	Y	8/8

JBI. Quasi-experimental studies (non-randomised experimental studies)										
Authors	Q1	Q2	Q3	Q 4	Q5	Q6	Q7	Q8	Q9	Score
(Assah et al., 2015)	Y	Y	Y	Y	Y	Y	Y	NC	Y	8/9
(Baumann et al., 2015)	Y	NC	Y	Y	Y	Y	Y	NC	Y	7/9

Mixed Methods Research (Qualitative and Quantitative)							
Authors	Q1	Q2	Q3	Q4	Q5	Q6	Score
Bayked et al., 2022)	Y	Y	Y	NC	NC	Y	4/6

Q, number of questions; Y, Yes; N, No; NC, Not clear

**Sources of JBI quality appraisal.**

Qualitative research (Lockwood et al., 2015).

Analytical cross-sectional studies (Moola, 2020).

Quasi-experimental studies (non-randomised experimental studies) (Tufanaru, 2020).

Mixed Methods Research (Qualitative and Quantitative) (Moorley & Cathala, 2019).
